# From Incremental Validity to Decision Utility: A Framework for Intelligence Testing in Education

**DOI:** 10.3390/jintelligence14060101

**Published:** 2026-06-05

**Authors:** Liliana Pedraja-Rejas, Carmen Araneda-Guirriman, Emilio Rodríguez-Ponce

**Affiliations:** 1 Departamento de Ingeniería Industrial y de Sistemas, Facultad de Ingeniería, Universidad de Tarapacá, Arica 1000000, Chile; caraneda@academicos.uta.cl; 2 Instituto de Alta Investigación, Universidad de Tarapacá, Arica 1000000, Chile; emilior.rodriguez.ponce@gmail.com

**Keywords:** intelligence testing, academic achievement, incremental validity, decision utility, educational placement, measurement comparability

## Abstract

Intelligence tests predict academic achievement, but their use in educational decision-making remains contested. We develop a decision-analytic framework, centered on a staged decision architecture, to determine when, for whom, and for which educational decisions intelligence testing adds value beyond grades, achievement measures, and contextual evidence. Drawing on psychometric scholarship, a generative account of achievement, and illustrative decision scenarios, we distinguish incremental validity from decision utility. Incremental validity refers to the predictive gain obtained by adding cognitive measures, whereas decision utility refers to the net benefit of using those measures once base rates, capacity constraints, error costs, fairness, and legitimacy are considered. We use the framework to identify conditions in which intelligence testing is expected to be most informative, especially educational transitions, contexts with uneven opportunity, and discrepancy-focused decisions such as underachievement or twice-exceptionality. We also specify minimum conditions for responsible use, including intended use, construct representation, reliability or precision, measurement comparability, predictive bias checks, and monitoring of distributional impact. We conclude that intelligence testing should be used conditionally and sequentially, with achievement and contextual indicators used first and cognitive assessment added only when it is likely to change the decision.

## 1. Introduction

The relationship between cognitive ability and academic achievement is among the most robust and enduring findings in differential psychology, intelligence research, and educational measurement. Early psychometric work provides the historical and conceptual point of departure for this research tradition: Spearman (1904) identified a general factor of intelligence that accounts for positive correlations across cognitive tasks, and later hierarchical accounts clarified how broad and specific abilities are organized within a general structure of cognitive functioning (Carroll, 1993). Contemporary intelligence research has continued to support the broad predictive reach of cognitive ability, including its relevance for educational outcomes across the life course (Deary, 2020; Lubinski, 2004). Across ages and educational stages, measures of general cognitive ability show moderate-to-strong associations with academic outcomes such as grades, standardized achievement scores, and educational attainment (Deary et al., 2007; Roth et al., 2015; von Stumm & Plomin, 2015). In postsecondary settings, standardized admissions tests and related cognitive measures have also demonstrated predictive and incremental validity for early college grade point average (GPA), persistence, and later academic performance, particularly when considered alongside prior academic record (Geiser & Santelices, 2007; Kuncel & Hezlett, 2007; Sackett et al., 2008).

At the same time, the psychometric case for intelligence testing does not rest on prediction alone. Within the core measurement tradition, the relevant questions also concern what test scores represent, how reliably they can be interpreted, and whether those interpretations are justified for their intended uses (AERA et al., 2014; Cronbach & Meehl, 1955; Messick, 1995). This point is especially important in education, where test scores are used not in the abstract but in concrete decisions about selection, placement, diagnosis, eligibility, and intervention. A measure may therefore be psychometrically strong in a general sense and still require separate justification for a specific educational use.

This distinction matters because educational decisions differ in purpose, stakes, and institutional constraints. Research on educational and psychological testing has long emphasized that evidence for score use must be evaluated in relation to the intended interpretation, target population, and decision context, rather than by predictive accuracy alone (AERA et al., 2014; Kane, 2013; Messick, 1995; Sireci & Benítez, 2023). A score that is useful for one purpose may add little to another. A cognitive measure may contribute to ranking under scarce capacity, yet have limited value in decisions centered on certifying current curriculum mastery. Likewise, it may be informative for clarifying discrepant learning profiles or underachievement while being unnecessary where grades and achievement data already provide a strong and sufficient signal. The value of intelligence testing in education is therefore not exhausted by the question of whether it predicts achievement. It also depends on how different sources of evidence map onto different decision purposes, what kinds of errors matter most in each case, and what fairness and legitimacy constraints bound acceptable use.

These issues have become more salient as the applied use of intelligence-related testing in educational decision-making has faced increasing resistance. A major contemporary driver has been the expansion of test-optional and test-blind admissions policies, accelerated by the COVID-19 pandemic and sustained in many settings thereafter. Although the evidence on these regimes remains uneven, their expansion has sharpened a broader debate about what cognitive and aptitude-related measures actually contribute to educational decisions, under what conditions, and at what cost (Camara, 2024; Gooch et al., 2024; McManus et al., 2023; Middleton et al., 2024; Osaki, 2022; Schultz & Backstrom, 2021). The resulting paradox is clear: cognitive measures continue to show meaningful predictive relationships with educational outcomes, yet their legitimacy and practical acceptability in educational decision-making have weakened.

Against this backdrop, we propose a decision framework for intelligence testing in education. Our core claim is straightforward: the key question is not whether intelligence tests predict achievement, but when, for whom, and for what educational decisions they add value beyond achievement measures and other available evidence. In that sense, the framework is intended as an extension of, not a substitute for, core psychometric principles. It begins from the premise that construct validity, reliability/precision, and intended-use arguments remain foundational. It then links two levels of analysis that are often treated separately: incremental validity, understood as the predictive gain obtained by adding cognitive measures over grades, achievement tests, and contextual indicators, and decision utility, understood as the net benefit of using those measures once real-world constraints are considered. This use-oriented view is consistent with contemporary validity scholarship, which treats test evaluation as inseparable from intended use and downstream consequences (Sinharay et al., 2025; Sireci & Benítez, 2023). It is also compatible with applied assessment work that conceptualizes utility in multi-criterion terms, extending beyond prediction to include impact, stakeholder acceptability, and resource costs (Divjak et al., 2023).

We make three main contributions. First, we offer a decision-centered framework that distinguishes predictive gain from decision utility and shows why the two should not be conflated. Second, building on a generative account of achievement, we specify the conditions under which intelligence testing is more or less likely to add educationally meaningful value. Third, and most directly for practice, we embed minimum psychometric, fairness, and legitimacy requirements into an implementable staged architecture designed to guide responsible use.

Methodologically, the framework is developed as a decision-analytic architecture rather than as a purely conceptual taxonomy: it specifies the components of decision utility, illustrates their operationalization under capacity constraints, translates them into staged decision rules, and derives falsifiable propositions for future empirical evaluation. The staged architecture is central to this contribution because it separates governance, baseline evidence, targeted cognitive assessment, and monitoring into distinct decision moments, thereby making conditional and sequential test use practically implementable.

To keep the argument analytically disciplined, the remainder of the article concentrates on three core domains in which the intelligence–achievement relation poses distinct inferential problems: (i) the identification and placement of high-potential students in gifted, accelerated, or advanced programs under scarce capacity, (ii) evaluations involving learning difficulties, specific learning disability (SLD), and intellectual disability (ID), and (iii) postsecondary admissions under test-required, test-optional, and test-blind regimes. Other applications are treated as extensions rather than as coequal domains. This narrowing is deliberate: it allows us to trade breadth for depth and to show more clearly how the same psychometric evidence changes meaning across decision purposes.

## 2. Conceptual Clarifications: Constructs and Decision Purposes

Debates about intelligence testing and academic achievement often suffer from construct slippage (treating intelligence, aptitude, achievement, grades, and authentic assessment as interchangeable measures of “ability”). A decision framework requires sharper distinctions.

### 2.1. Intelligence and Aptitude

Intelligence, particularly general cognitive ability (g), is commonly understood as a broad capacity for reasoning, learning, and solving problems in ways that transfer across tasks (Carroll, 1993; Spearman, 1904). In this paper, the term intelligence testing is used broadly to encompass both measures of general cognitive ability and, in some educational contexts, aptitude measures. The common thread is that these tests are used to infer learning potential, reasoning efficiency, or readiness to acquire new knowledge, rather than mastery of curriculum already attained (Deary, 2020; Lubinski, 2004; Snow, 1992).

For educational decision-making, the key issue is not only the abstract construct, but the level at which score interpretations are made. Intelligence may be represented through a general factor, broad cognitive abilities, or composite scores, but these levels are not interchangeable for interpretive purposes (Schneider & McGrew, 2012). Accordingly, the relevant question is not how many scores an instrument produces but which level of score can validly support the educational inference at stake (Carroll, 1993; Messick, 1995; Schneider & McGrew, 2012).

### 2.2. Achievement

Achievement reflects acquired knowledge and skills, typically indexed by curriculum-aligned tests, course performance, or credential outcomes. Achievement is strongly correlated with intelligence, but not redundant, because it is a cumulative outcome shaped not only by cognitive capacity but also by instructional exposure and opportunity structures (Ceci, 1991; Deary et al., 2007; Rohde & Thompson, 2007). A generative view of achievement therefore treats it as partly realized potential under specific educational conditions rather than as a pure index of learning capacity alone.

### 2.3. Grades

Grades aggregate evaluation across time and contexts and often predict later academic outcomes (Geiser & Santelices, 2007). However, grades also embed school-level variation in standards, course rigor, and grading practices, and therefore mix information about performance with contextual opportunity (Koretz, 2017). Evidence further suggests that the meaning of a given GPA can vary across high schools, complicating assumptions that grades are commensurate indicators of readiness across settings (Allensworth & Clark, 2020).

### 2.4. Authentic Assessment

Authentic assessment emphasizes contextualized performance tasks, such as portfolios, projects, or other demonstrations of applied competence intended to approximate real-world performance (Wiggins, 1998). Its main strength is that it can increase ecological validity and instructional alignment. Its limitation, especially in high-stakes comparative settings, is that it is often harder to standardize across raters, tasks, and contexts, which can complicate reliability and comparability at scale (Koretz, 2017). Within the present framework, authentic assessment is therefore most relevant when the decision purpose is to evaluate current mastery or applied performance, rather than to infer broader learning potential under comparative selection conditions.

### 2.5. Decision Purposes: Why the Same Measure Can Be “Good” or “Bad” Depending on the Goal

Measurement utility is conditional on decision purpose. Building on validity and assessment-use frameworks that treat test interpretation as inseparable from intended decision purpose, target population, and consequences of use, we distinguish four common purposes (AERA et al., 2014; Kane, 2013; Messick, 1995).

First, tests are used for ranking and selection (e.g., admissions or scholarships), where the primary goal is to optimize prediction under explicit capacity constraints. Second, tests are used for placement (e.g., high-potential programs, tracking, or remediation), where the central objective is person–environment fit, identifying who is most likely to benefit from a given instructional setting or program. Third, tests support diagnosis and eligibility decisions (e.g., specific learning disability [SLD], intellectual disability [ID], etc.), where the aim is to clarify a learner’s profile and to rule in or rule out categories that trigger access to services. Fourth, tests inform intervention planning, where the relevant evidence is the information that most directly supports the selection of supports that can change outcomes; in these cases, actionable profiles may matter more than broad predictive accuracy.

Table 1 summarizes the corresponding implications for evidence use and highlights the dominant error-cost concerns associated with each purpose.

## 3. A Generative Model of Academic Achievement

To formalize when intelligence testing adds information beyond achievement measures, we adopt a generative view in which achievement reflects the joint operation of multiple interacting components over time:

Achievement_(t)_ = f (Learning capacity, Knowledge_(t−1)_, Opportunity to learn, Noncognitive influences). (1)

In this model, capacity refers to learning efficiency: cognitive ability influences how rapidly and deeply students acquire knowledge and skills given instructional inputs and task demands (Carroll, 1993; Deary et al., 2007). Accumulated knowledge captures the cumulative nature of learning; prior achievement is often a strong proximal predictor because it indexes stored knowledge, practiced skills, and successful task performance under earlier conditions (Rohde & Thompson, 2007). Opportunity to learn represents the structural conditions that enable capacity to translate into knowledge, such as curriculum access, instructional quality, resources, and stability, so that unequal opportunity can weaken the correspondence between achievement and underlying capacity (Ceci, 1991). Finally, noncognitive influences, including motivation, self-regulation, conscientiousness, persistence, health/stress, and perceived value, can shape achievement trajectories by affecting engagement, persistence, the use of available learning opportunities, and the accumulation of knowledge over time (Credé et al., 2017; Duckworth et al., 2007; Theobald, 2021; Xu et al., 2023).

This view is consistent with research on motivation and self-regulated learning, which shows that students’ motivational beliefs, engagement, strategy use, and resource management shape how they use available opportunities and translate prior knowledge into subsequent performance (Theobald, 2021; Xu et al., 2023). A key implication of this generative account is that intelligence and achievement, while correlated, occupy different nodes in the achievement-generating system. Intelligence measures tend to capture learning capacity, learning efficiency, and adaptation to future demands, whereas achievement measures primarily capture realized outcomes under prior instructional, opportunity, and motivational conditions. This distinction clarifies why intelligence testing can be informative even when achievement data are available: the two sources of evidence are related but not redundant, and their relative utility depends on the decision context and on how opportunity, instruction, and noncognitive conditions have shaped observed performance.

**Figure 1:**
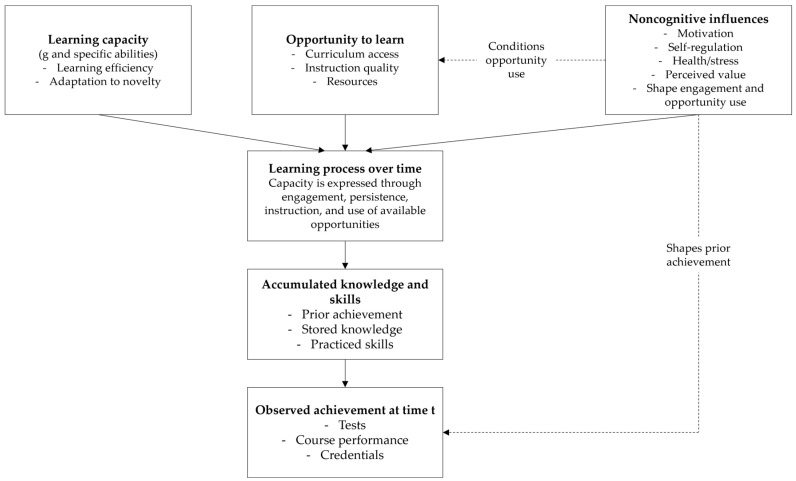
Generative model of academic achievement.

Figure 1 depicts achievement at time *t* as a realized outcome generated over time through the interaction of learning capacity, opportunity to learn, noncognitive influences, and accumulated knowledge and skills. Learning capacity refers to general and specific cognitive abilities, learning efficiency, and adaptation to novelty, whereas opportunity to learn captures curriculum access, instructional quality, and resources. Noncognitive influences, such as motivation, self-regulation, health/stress, and perceived value, are treated as conditions that shape how capacity is expressed, how opportunities are used, and how knowledge accumulates over time. The model highlights why intelligence and achievement are correlated but non-redundant: achievement reflects realized outcomes under prior learning conditions, whereas cognitive ability more directly indexes learning capacity and adaptation to future demands.

## 4. The Decision Utility and Incremental Validity Framework

The decision value of intelligence testing depends not only on whether it predicts academic outcomes but on whether it adds information beyond what is already available and whether that added information improves real decisions under constraints. This section introduces an integrated framework that links incremental validity, the predictive gain obtained by adding cognitive measures to existing evidence such as grades, achievement tests, and contextual indicators, to decision utility, defined as net benefit once base rates, capacity limits, and the costs of false positive and false negative decisions are considered. The equations and simulations used below are illustrative decision-analytic tools, not empirical models estimated in this article; they clarify how predictive gain, base rates, error costs, and capacity constraints can alter the value of adding cognitive information.

### 4.1. Incremental Validity: Prediction Gains over Existing Evidence

The standard psychometric question, “Does intelligence predict achievement?”, is insufficient for many educational decisions in which grades, achievement measures, and contextual indicators are already available. However, the question of incremental validity is only meaningful after a prior threshold has been met: the score interpretation must be psychometrically defensible for the intended use. In other words, a test cannot be said to “add value” in any meaningful sense unless there is already adequate support for the construct representation of the score, for its reliability/precision at the relevant level of interpretation, and for the appropriateness of using that score in the target population and decision context (AERA et al., 2014; Cronbach & Meehl, 1955; Messick, 1995).

Once that threshold is met, the relevant criterion becomes incremental validity, defined as the predictive gain achieved by adding intelligence measures to the existing evidence base:

ΔR^2^ = R^2^(Achievement ∣ GPA, Achievement tests, Context, Intelligence) − R^2^(Achievement ∣ GPA, Achievement tests, Context) > 0. (2)

Evidence from selection contexts indicates that cognitive measures often retain incremental validity over prior academic record, particularly at key educational transitions (Kuncel & Hezlett, 2007; Sackett et al., 2008). At the same time, incremental validity is inherently context-dependent. It can vary with range restriction (e.g., selective samples), heterogeneity in grading standards, and opportunity inequality that differentially shape the meaning of observed achievement across educational settings.

Framed this way, incremental validity is not a standalone psychometric virtue. It is a conditional property of an already interpretable score used for a justified purpose. A measure may show a statistically nontrivial gain over existing predictors and still remain inappropriate if the score lacks sufficient precision for the stakes involved, if the interpretation is weakly supported for the relevant population, or if the intended use itself has not been adequately justified. The practical implication is that predictive gain should be evaluated only after the more basic question has been answered: is this test appropriate for this use, in this setting, for this population?

### 4.2. From Prediction to Decisions: Why Validity Is Not Utility

A measure can improve prediction yet have limited practical value if it does not change decisions, or if its costs and distributive consequences dominate its benefits. Building on the actuarial decision tradition (Dawes et al., 1989; Meehl, 1954), we define decision utility as the net expected benefit of using a measure under real-world constraints:

Decision utility = f (Predictive gain, Base rates, Error costs, Capacity constraints, Distributional impact, Legitimacy).(3)

The equation is not intended to reduce educational decision-making to a single universal metric. Rather, it specifies the components that must be made explicit before intelligence testing can be judged useful for a given decision. Predictive gain, base rates, error costs, and capacity constraints can be examined through decision-analytic metrics such as net benefit, whereas distributional impact and legitimacy require separate subgroup and institutional evaluation.

This distinction matters because educational systems operate under unavoidable constraints. Seats in programs for high-potential students are limited, diagnostic evaluations are costly, and admissions decisions are high stakes. Moreover, perceived legitimacy, among students, families, educators, and the broader public, shapes compliance, institutional trust, and the sustainability of any assessment practice.

The framework specifies six components that jointly determine whether adding intelligence testing is decision-relevant. First, predictive gain concerns incremental validity over the existing evidence base and the extent to which added prediction is practically meaningful. Second, base rates matter because the prevalence of the target condition (e.g., SLD, high potential) shapes the expected proportion of false positives and false negatives, even when tests perform well on average. Third, the error-cost structure conditions utility: false positives can misallocate scarce resources or create person–program mismatch, whereas false negatives can deny opportunity or support, often with cumulative downstream consequences. Fourth, capacity constraints (e.g., limited program seats, diagnostic resources, or support services) further shape thresholds and the trade-offs institutions can realize in practice. Fifth, distributional impact captures how decisions affect subgroup outcomes (e.g., access, representation, and downstream attainment) and therefore requires explicit monitoring rather than assumption. Finally, legitimacy bounds acceptable use through perceived fairness, transparency, and stakeholder trust; responsible practice therefore requires minimum standards for measurement comparability and predictive bias checks, alongside clear communication and ongoing auditing of consequences.

#### Decision-Curve Analysis and Net Benefit

Decision utility can be evaluated directly using decision-analytic metrics that translate prediction into expected value under explicit thresholds. A widely used approach is decision-curve analysis (DCA), which compares alternative decision rules by their net benefit across a range of threshold probabilities (Vickers & Elkin, 2006; Vickers et al., 2016).

Let p_t_ denote the threshold probability at which the decision-maker is indifferent between acting and not acting (e.g., allocating a seat, triggering an evaluation, or offering support). In the simplest setting, p_t_ summarizes the relative cost of false positives versus false negatives. For a given rule, net benefit at threshold p_t_ can be written as:

Net benefit = (TP/N) − (FP/N) × [p_t/_(1 − p_t_)], (4)

where TP and FP are the numbers of true and false positives generated by the rule among N cases. The weighting term p_t_/(1 − p_t_) formalizes the implicit exchange rate between false positives and true positives at threshold p_t_. Importantly, net benefit is sensitive to (i) base rates (prevalence of the target condition), (ii) error-cost ratios (which determine p_t_), and (iii) institutional capacity constraints K (which often impose a fixed number of admits/placements/support slots).

In educational settings, DCA clarifies why a small gain in incremental validity can be either consequential or negligible: when p_t_ is extreme (very scarce capacity or very high false-positive costs) and/or base rates are low, a modest change in ranking around the operational margin can meaningfully shift who is selected and the resulting utility. Conversely, when decisions are already near-saturated by existing evidence (strong baseline models), additional testing may not change actions and can lower net benefit once financial, time, and legitimacy costs are incorporated.

Accordingly, the framework recommends reporting not only predictive metrics (e.g., area under the receiver operating characteristic curve [AUC], Brier score, calibration) but also utility metrics (net benefit and cost-adjusted net benefit) under plausible ranges of p_t_, base rates, and capacity K for the intended decision context. Importantly, within the present framework, DCA operationalizes only one part of Equation (3): it captures how predictive gain translates into threshold-sensitive utility under base rates, error costs, and capacity constraints, but it does not by itself resolve distributional impact or legitimacy. Those dimensions require separate evaluation at the subgroup and institutional levels.

### 4.3. Stylized Worked Example of Decision Utility Under Capacity Constraints

To show how the decision-analytic component of the framework can be operationalized, this section presents a stylized worked example of a capacity-constrained admissions setting. The aim is not to provide empirical validation of intelligence testing, but to demonstrate how specific assumptions about base rates, error costs, and capacity constraints change the utility of adding cognitive information. Consider an admissions decision with N = 1000 applicants and K = 200 available seats. Let Y = 1 denote “success” (e.g., persistence to year 2 or first-year GPA above a threshold). The institution must choose a decision rule that improves expected utility while remaining subject to fairness and legitimacy constraints.

#### 4.3.1. Setup

Assume each applicant i has a predicted probability of success p_i_ = P(Y_i_ = 1 ∣ evidence). The institution assigns benefit B = 1 to a true positive (admit a student who succeeds), cost C = 2 to a false positive (admit a student who does not succeed), and cost L = 0.5 to a false negative (reject a student who would succeed). The expected utility of admitting is

U(admit ∣ p_i_) = p_i_⋅B − (1 − p_i_)⋅C, (5)

and the expected utility of rejecting is

U(reject ∣ p_i_) = − p_i_⋅L. (6)

Admitting is preferred when

ΔU(p_i_) = U(admit ∣ p_i_) − U(reject ∣ p_i_) = p_i_ (B + C + L) − C > 0, (7)

which implies the threshold probability

p_t_ = C/(B + C + L). (8)

With these values, p_t_ ≈ 0.571. Because ΔU(p_i_) is monotonic in p_i_, ranking applicants by predicted utility is equivalent here to ranking them by predicted probability. However, because capacity binds (K = 200), the realized operational cutoff is determined not only by the threshold itself but also by how each evidence regime reshapes the top of the ranking near the admission margin.

We compare two evidence regimes:

- Achievement-only: p_i_ = P(Y = 1 ∣ grades/achievement record)
- Achievement + test: p_i_ = P(Y = 1 ∣ grades/achievement record + cognitive test)

Under both regimes, applicants are ranked by p_i_, and the top K are admitted.

#### 4.3.2. Illustrative Simulation Across Decision Conditions

Table 2 reports two stylized scenarios. Scenario A assumes a moderate base rate of success in the applicant pool. Scenario B assumes a lower base rate, which increases the false-positive burden and reduces attainable net benefit under the same threshold and seat constraint. In both scenarios, the question is whether adding test information changes ranking enough at the operational margin to improve expected utility.

Because K is fixed and total positives are scenario-specific constants, net benefit provides a compact decision-analytic summary of the same trade-off defined above. In this setting, utility-improving regimes are those that improve the true-positive/false-positive balance at the admission margin under the stated threshold. The table therefore does not estimate a predictive model; it reports illustrative expected classification outcomes under two evidence regimes while holding N, K, and p_t_ constant.

The worked example illustrates three points. First, when capacity is fixed, even modest predictive gain can improve outcomes by changing who is admitted near the operational cutoff. In Scenario A, adding test information increases expected true positives and reduces false positives at the same seat constraint, thereby raising net benefit. Second, the same predictive gain can matter differently depending on the surrounding decision environment. In Scenario B, where the base rate is lower, both regimes perform worse because the false-positive burden is larger; adding the test still improves expected utility, but the gain is smaller and may remain insufficient to justify routine testing. Third, the relevant question is therefore not whether a test predicts in general but whether it improves the decision system under the actual combination of prevalence, cost asymmetry, and capacity limits.

#### 4.3.3. Distributional Impact Is Not Implied by Net Benefit

Net benefit does not exhaust the utility function in Equation (3). Distributional impact remains a separate evaluative dimension because a regime can improve average utility while still shifting subgroup outcomes in ways that require independent scrutiny. Let G1 and G2 denote two applicant groups (e.g., advantaged and constrained-opportunity applicants). In some settings, adding test information may increase access for applicants whose achievement record underestimates future learning potential; in others, it may reinforce existing disparities if access to preparation, accommodations, or score reporting is unequally distributed. For that reason, subgroup outcomes should be treated as empirical targets of evaluation (admit rates, persistence, graduation, and downstream attainment by group) rather than inferred from predictive performance alone.

#### 4.3.4. Legitimacy Constrains Feasible Regimes

Legitimacy is likewise not reducible to net benefit. Even when a regime improves expected utility, it may remain infeasible if it is perceived as opaque, unfair, or inconsistent with institutional commitments or legal standards. In practice, legitimacy constraints require transparent decision rules, minimum psychometric gatekeeping, and ongoing auditing of both predictive performance and distributional consequences. Legitimacy therefore enters the framework not as a rhetorical add-on but as a practical constraint on which utility-improving regimes are actually implementable.

#### 4.3.5. Implications for the Framework

This stylized worked example clarifies why competing evidence regimes should be evaluated as decision systems rather than as isolated measurement choices. The central empirical question is not simply whether adding a test increases prediction, but whether it improves expected utility under the relevant combination of base rates, error costs, capacity constraints, distributional consequences, and legitimacy conditions. The worked example therefore operationalizes only the decision-analytic component of Equation (3), while the broader framework requires that any utility gain also be evaluated for subgroup impact and institutional feasibility. This is precisely the type of evaluation the proposed framework is designed to structure.

### 4.4. When Intelligence Testing Should Add the Most (and the Least)

Based on the generative model in the previous section, intelligence testing is expected to add the most decision-relevant information in conditions where prior achievement is a weaker proxy for future learning efficiency or where observed performance is strongly shaped by unequal opportunity. These expectations follow from evidence that prior achievement and cognitive measures overlap but are not redundant, and that the meaning of achievement indicators can vary with instructional exposure, grading standards, and opportunity structures (Allensworth & Clark, 2020; Ceci, 1991; Deary et al., 2007; Rohde & Thompson, 2007). High-utility contexts therefore include educational transitions, where new task demands reduce the generalization of past achievement; settings with uneven opportunity, where achievement may underrepresent capacity; and discrepant profiles, such as underachievement, twice-exceptionality, or otherwise complex learning profiles. Intelligence testing may also be especially informative for decisions involving high cognitive-load programs, including fast-paced, cumulative curricula where learning efficiency and adaptation to future demands are central.

By contrast, intelligence testing is expected to have low utility in stable environments where achievement provides a strong and sufficient signal, in decisions aimed primarily at certifying current mastery, in settings where any incremental validity would not materially change decisions, or in contexts where minimum fairness requirements cannot be met. Table 3 summarizes high- and low-expected-utility conditions, recommended decision designs, and the minimum standards required for responsible use.

### 4.5. A Staged Decision Architecture

A practical implication of the framework is that the default decision design should often be sequential, rather than relying on any single indicator from the outset. This follows directly from the distinction between validity and utility: when grades, achievement measures, and contextual information already provide a strong signal, adding cognitive testing everywhere is unlikely to improve decisions and may increase costs and distributive concerns. This staged logic is consistent with assessment-use principles that require the evidentiary burden and intrusiveness of testing to be proportional to the decision stakes and to the expected contribution of the score to the decision (AERA et al., 2014; Messick, 1995; Sireci & Benítez, 2023). Instead, the framework points to a staged architecture that concentrates measurement effort where it can plausibly change conclusions.

In this architecture, Stage 0 specifies the decision purpose, capacity constraint, fairness constraints, and error-cost assumptions before any testing is deployed. Stage 1 establishes the baseline evidence model using achievement, grades, and contextual indicators. Stage 2 asks whether cognitive information has sufficient expected value for the decision to justify additional testing, especially in uncertain, borderline, or discrepant cases. Stage 3 links the final decision to monitoring of predictive performance, distributional impact, and perceived legitimacy. The staged architecture therefore translates the components of decision utility into an operational sequence: first clarify the decision problem, then evaluate the baseline evidence, then add cognitive testing only when it can change the decision, and finally monitor whether the decision system remains defensible over time.

Accordingly, the framework recommends a staged process in which decision-makers begin with evidence that is widely available and directly tied to educational performance. In Stage 1 (broad screening), this includes transcripts/GPA, achievement measures, and relevant context indicators. Stage 2 (targeted cognitive assessment) is reserved for cases in which the decision remains uncertain and in which intelligence information could plausibly change the conclusion, such as borderline selection, placement questions, or discrepant profiles requiring clarification. In Stage 3 (decision + monitoring), the institution formalizes decision rules with explicit assumptions about error costs and then monitors predictive performance, distributional impact, and perceived legitimacy to support periodic recalibration.

**Figure 2:**
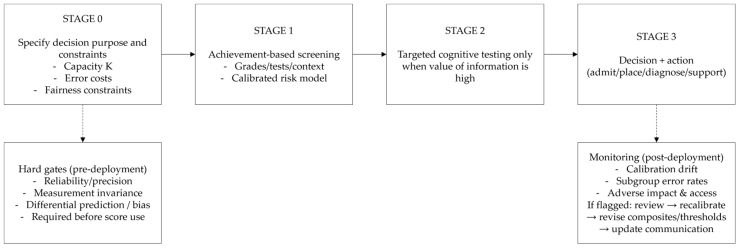
Staged decision architecture for intelligence testing in education.

Figure 2 summarizes this logic visually and clarifies how each stage maps onto the utility framework. By treating cognitive assessment as a targeted second-stage input rather than a blanket requirement, the architecture preserves incremental value where it matters most while minimizing cost, unnecessary testing exposure, and over-reliance on any single indicator.

## 5. Minimum Standards for Responsible Use

A decision framework for intelligence testing must specify not only when cognitive information adds predictive value, but also when its use is permissible. Consistent with the Standards for Educational and Psychological Testing, minimum standards should not be treated as optional complements to prediction. They define whether score use is defensible for a particular purpose, population, and decision context (AERA et al., 2014). Responsible use therefore begins with intended use, construct representation, and reliability/precision; only then should institutions evaluate comparability, predictive bias, distributional impact, and legitimacy.

Within the staged architecture developed above, these standards operate as admissibility and continuation conditions. They determine whether score use can begin, whether targeted cognitive assessment can be added, and whether implemented score use should be maintained, revised, or suspended. In this sense, minimum standards convert the architecture from a procedural sequence into a regulated decision system.

### 5.1. Intended Use, Construct Representation, and Reliability/Precision

Before a score is used in educational decision-making, there must be a clear rationale for why the test is being used, what construct the score is intended to represent, and how that interpretation contributes to the specific decision at hand (AERA et al., 2014). This requirement is prior to statistical performance. A score may predict later outcomes and still be poorly justified for a particular use if the construct being interpreted is underspecified, if the score combines heterogeneous components without a defensible rationale, or if the inference exceeds what the test can support.

Reliability and precision must also be evaluated in relation to the intended interpretation, not as generic properties of the instrument. The relevant question is whether the total score, subscore, or composite actually used in the decision is sufficiently precise for the stakes involved and for the domain over which the score is meant to generalize. Lower precision may be more tolerable in low-stakes or reversible decisions when corroborating evidence is available. In higher-stakes or less reversible decisions, the evidentiary burden is stronger.

This distinction is especially important when intelligence testing is used for more than broad screening. Evidence that a battery yields a defensible global score does not automatically justify interpretation of factor scores, profile discrepancies, or subtest scatter. Broad ranking or screening decisions may sometimes be supported by composite scores, whereas individualized explanatory claims require tighter construct-to-decision matching and should not rest on profile reading by default (Cronbach & Meehl, 1955; Sinharay et al., 2025; Sireci & Benítez, 2023). If intended use, construct representation, and reliability/precision are not established, subsequent claims about incremental validity or decision utility are not yet interpretable.

### 5.2. Measurement Comparability and Predictive Bias

Once the intended use and score interpretation are justified, institutions must evaluate whether the score has equivalent meaning across relevant groups. Before scores are used for high-stakes comparative decisions, evidence should support that the instrument measures the same construct in the same way across populations that may differ in language background, educational opportunity, disability status, or other characteristics relevant to score interpretation (Meredith, 1993). Comparability must be examined at the score level that actually drives the decision. Evidence for a global score does not automatically imply that every subscore, discrepancy, or profile contrast is equally comparable across groups.

Responsible use also requires evaluation of predictive bias in the local decision context. The differential prediction tradition defines test bias as a prediction problem: whether a test systematically over- or under-predicts outcomes for groups given the same score (Cleary, 1968). Accordingly, institutions should examine calibration, prediction errors, and subgroup false-positive and false-negative rates. A score may be coherent at the construct level and still function unevenly in prediction across subpopulations, making subgroup analysis essential for responsible use.

### 5.3. Selection Errors, Adverse Impact, and Explicit Trade-Offs

Even when tests are interpretable, sufficiently precise, comparable, and free from predictive bias, subgroup mean differences can still produce differential selection rates. The relationship among validity, bias, selection errors, and adverse impact depends on base rates, cut scores, capacity constraints, and the structure of the decision rule (Aguinis & Smith, 2007). The applied literature has also emphasized the diversity-validity dilemma and the role of structured multi-measure systems in managing this tension (Ployhart & Holtz, 2008).

The framework therefore requires institutions to make trade-offs explicit: What outcomes are optimized? What error costs are acceptable? Which fairness constraints are binding? Absent explicit trade-offs, systems risk either (a) maximizing prediction while ignoring distributional consequences or (b) adopting procedural or low-information reforms that reduce information without reliably improving equity.

### 5.4. Transparency, Monitoring, and Remediation

Because educational decisions are socially embedded, responsible use also depends on transparency and legitimacy. Institutions should communicate the purpose of testing, the role of scores in the decision, the availability of appeals, and the safeguards against misuse. Legitimacy is not external to psychometrics; it is one of the conditions under which psychometric evidence can be translated into acceptable institutional practice (AERA et al., 2014; Messick, 1995; Sireci & Benítez, 2023).

Governance should therefore distinguish pre-deployment requirements, ongoing monitoring, and remediation. Before deployment, score use should not proceed unless the intended interpretation is justified, reliability/precision is adequate, comparability has been examined where relevant, and the decision design specifies thresholds, capacity constraints, and error-cost assumptions. Once implemented, institutions should monitor calibration drift, subgroup error rates, distributional consequences, and indicators of perceived legitimacy such as appeals, complaints, or stakeholder distrust.

When deterioration, inequitable impact, or loss of legitimacy is detected, remediation should be mandatory rather than discretionary. Responses may include recalibrating thresholds, revising score use, narrowing the decision context, improving access conditions, or suspending score use until re-validation supports reinstatement. Under this framework, responsible practice is defined not by whether a test predicts in the abstract, but by whether the decision system remains psychometrically defensible, fair in operation, and legitimate over time.

## 6. Implications for Educational Decisions

This section outlines the practical implications of the proposed framework for educational decision-making across three primary application fields: high-potential student identification and placement, learning difficulties and SLD/ID evaluation, and postsecondary admissions. These domains represent distinct decisional logics—person–environment fit under capacity constraints, diagnostic/intervention-oriented evaluation, and institutional selection under changing testing regimes.

### 6.1. Identification and Placement of High-Potential Students

The identification and placement of high-potential students in gifted, accelerated, or advanced programs are prototypical cases of person–environment fit under capacity constraints. The relevant question is not whether cognitive ability predicts academic success in general, but whether adding cognitive evidence improves the identification of students who are likely to benefit from accelerated pace, abstract instruction, or specialized programming beyond what can already be inferred from achievement and school performance alone. This issue becomes especially important when achievement may underrepresent potential because of unequal opportunity to learn, uneven instructional exposure, or contextual barriers that suppress the observed record.

This view aligns with recent work in gifted education and talent identification, which emphasizes that identification systems should be evaluated not only by whom they select, but also by how well they align with available programming, access conditions, and equity goals (Peters, 2022; Peters et al., 2024; Stambaugh et al., 2024). Recent evidence also cautions that identifying students as gifted is insufficient if the services they receive are weakly aligned with the domains or learning needs for which they were selected (Siegle et al., 2025). In this sense, high-potential student identification is not simply a labeling problem but a decision about whether a learner is likely to benefit from a specific educational environment.

Within the proposed framework, cognitive testing is most defensible in high-potential student identification and placement decisions when it changes the inference in educationally meaningful ways. This is most likely in discordant or borderline cases, especially when strong reasoning potential is not yet fully visible in grades or achievement indicators, or when the program being considered places unusual demands on learning pace and abstraction. A staged architecture is therefore preferable to blanket testing: broad screening can begin with achievement, school record, and contextual evidence, followed by targeted cognitive assessment when uncertainty remains or when the existing record risks missing high-potential students. In this domain, false negatives are especially consequential because they can lock students out of opportunity, while false positives can create person–program mismatch and dilute limited resources. The framework therefore supports selective, decision-focused use of cognitive evidence rather than universal first-stage testing.

### 6.2. Learning Difficulties, SLD, and ID Evaluation

In evaluations involving learning difficulties, SLD, and ID, decisions are typically explanatory and intervention-oriented: why is academic growth not occurring as expected, what interpretation best fits the learner’s profile, and what supports are most likely to help? Reviews emphasize that SLD identification practices vary widely and that psychometric evidence can be interpreted differently across diagnostic and educational eligibility frameworks (McGill et al., 2020). More recent work discusses how assessment practices for SLD and ID can be aligned with contemporary classification systems while remaining focused on actionable educational planning (Fletcher & Miciak, 2024).

This position is consistent with recent debates in SLD and ID assessment, which caution against interpreting cognitive profiles as automatically instructional or diagnostic unless they change classification, differential diagnosis, or intervention planning (Fletcher & Miciak, 2024; McGill et al., 2020). Within the proposed framework, cognitive assessment is therefore most defensible when it is genuinely decision-relevant, that is, when it changes the classification conclusion, clarifies a differential diagnosis, or alters the intervention plan. This is most plausible when the evaluation concerns broad intellectual impairment versus more specific learning difficulties, when the profile is complex or discrepant, or when convergent evidence is needed to interpret ambiguous achievement and response-to-instruction data. By contrast, the framework cautions against treating comprehensive cognitive batteries as intrinsically informative simply because they generate detailed profiles.

The field’s own measurement debates reinforce this position. Recent reviews emphasize that SLD and ID are among the few educational classifications that explicitly depend on psychometric test performance, yet the inferential role of cognitive assessment differs across them. For ID, global ability evidence enters alongside adaptive functioning and developmental history; for SLD, by contrast, detailed cognitive profiling is far more controversial and does not automatically imply instructional relevance. A decision framework therefore shifts the question away from whether cognitive testing is available and toward whether the score interpretation changes differential diagnosis, exclusion of alternative explanations, or intervention planning in ways that exceed what can already be learned from achievement data and instructional response (Fletcher & Miciak, 2024).

### 6.3. Postsecondary Admissions: Test-Required, Test-Optional, and Test-Blind

The postsecondary admissions domain provides one of the clearest illustrations of why validity debates alone do not resolve educational policy decisions. In this setting, the central question is not simply whether standardized tests predict academic achievement, but how different admissions regimes change the information available to institutions, the incentives facing applicants, and the structure of decision error under explicit constraints of fairness, legitimacy, and institutional capacity. The evidence based on test-optional and test-blind admissions remains uneven in maturity. Descriptive reports, field analyses, and policy commentaries are especially useful for documenting the rapid diffusion of these regimes and the heterogeneity of their implementation, whereas causal and review-based evidence on enrollment, graduation, and equity effects remains comparatively emergent (Camara, 2024; Middleton et al., 2024; Schultz & Backstrom, 2021). This matters because optionality is not a trivial procedural variation. Under optional submission, applicants may selectively disclose scores, thereby changing the evidentiary environment within which admissions decisions are made and potentially shifting the distribution of decision errors (McManus et al., 2023).

The more policy-outcome-oriented evidence remains mixed and still developing. Quasi-experimental analyses suggest modest average increases in underrepresented minority enrollment, alongside heterogeneous effects on graduation outcomes that vary by institutional selectivity (Osaki, 2022). Policy design also varies substantially across institutions, including differences between test-optional and test-blind regimes and extensions of these policies to scholarships and related decisions.

Recent evidence indicates that enrollment effects differ not only by institutional selectivity but also by how optionality is specifically operationalized (Rosinger et al., 2024). A review of the emerging literature similarly concludes that test-optional policies do not benefit equity in all contexts and therefore are unlikely to function as a standalone equity intervention across settings (Gooch et al., 2024). More recent work helps explain this heterogeneity, suggesting that non-uniform diversity outcomes can arise because institutions may retain strong underlying preferences for standardized tests even after removing formal requirements, and because competing institutional priorities can attenuate equity-related effects (Hsu & Sharkey, 2025). Recent applicant-level evidence from the Common Application also suggests that test-optional adoption did not produce sustained increases in applications to the most selective colleges, including among underrepresented groups, even as those colleges enrolled more lower-scoring students with strong grades, particularly first-generation and lower-income students; the same study also reports increased enrollment of higher-scoring students at less selective colleges, raising the possibility of greater undermatching under some conditions (Avery et al., 2025).

At the same time, preliminary institutional and multi-institution evidence suggests that test scores may remain informative for predicting early academic performance and may provide a contextual signal for applicants from constrained-opportunity backgrounds, especially when combined with grades and high school context indicators (Bastedo et al., 2023; Cascio et al., 2024; Friedman et al., 2024). This is broadly consistent with recent validation syntheses of the SAT and ACT: while these tests are often characterized as psychometrically strong, the validity of high-stakes admissions decisions derived from them, particularly for racially minoritized and socioeconomically disadvantaged students, remains a point of active contention (Amrein-Beardsley et al., 2025).

Taken together, these findings suggest that the consequences of admissions optionality hinge less on the validity of any single measure than on how competing regimes reshape information availability, applicant incentives, and institutional decision rules. Viewed through the present framework, test-required, test-optional, and test-blind regimes should therefore be evaluated as constrained decision systems rather than as isolated measurement choices. Test-blind regimes can remove a potentially informative signal for some applicants and institutions, thereby shifting greater weight to other indicators, such as school-specific grading, course access, or contextual records, whose psychometric and fairness properties may differ. Test-optional regimes, by contrast, alter the information environment through self-selection into disclosure. This is also consistent with earlier findings showing that high-school GPA may provide a stronger and more stable signal than standardized tests in some admissions contexts (Allensworth & Clark, 2020; Geiser & Santelices, 2007). The framework implication is therefore not that testing is categorically useless but that a universal first-stage testing requirement needs additional justification when transcript-based evidence already carries much of the stable signal. The more defensible use case is often targeted or sequential: test information should be added when uncertainty is high, when misclassification costs are asymmetric, or when institutions can demonstrate incremental net benefit under explicit fairness constraints. The practical implication is not that one regime is always superior, but that each regime should be compared on utility grounds in the relevant institutional context.

### 6.4. An “If-Then” Translation for Practice

To support implementation, the framework can be translated into a simple if–then logic that links evidence use to decision-making purposes. Rather than relying on a single rule across contexts, schools and universities should begin by specifying what kind of decision is being made (selection, placement, diagnosis/eligibility, or intervention planning) and then ask whether cognitive testing is likely to change that decision in a meaningful way beyond grades, achievement, and contextual evidence. Where the decision concerns current curriculum mastery or applied performance, curriculum-aligned achievement measures and authentic assessment may be more informative than cognitive testing.

The checklist is intended to operationalize the framework at the institutional level. It identifies the minimum decision record that should exist before cognitive testing is used, the conditions under which testing should be avoided, and the monitoring evidence needed once a testing regime is implemented. Table 4 summarizes this logic as an implementation checklist for practice.

## 7. Falsifiable Propositions and Research Agenda

The framework yields testable predictions about when intelligence testing should, and should not, add decision-relevant information. We therefore articulate four falsifiable propositions that connect the generative model and the decision-utility framework to observable empirical patterns. These propositions are not intended as exhaustive hypotheses. Rather, they specify where the framework’s core claims are empirically vulnerable: whether incremental validity increases under transition and opportunity-inequality conditions, whether staged testing improves utility relative to blanket testing, and whether testing-policy regimes change decision errors through information availability and strategic disclosure.

P1 (Transitions) predicts that the incremental validity of intelligence will be larger for outcomes in novel environments (e.g., first-year postsecondary performance) than for outcomes in stable environments. P2 (Opportunity inequality) predicts that intelligence will add more value where prior achievement is more strongly confounded with opportunity structures, such that achievement underrepresents underlying learning capacity. P3 (Sequential architecture) predicts that staged screening combined with targeted cognitive assessment will yield comparable or improved decision utility relative to blanket testing, operationalized through net benefit, error rates at the operational margin, cost, and testing exposure. P4 (Policy regimes) predicts that test-optional policies increase strategic disclosure and that the magnitude of information loss should predict shifts in error distribution, varying by institutional selectivity and implementation design (McManus et al., 2023; Rosinger et al., 2024).

These propositions can be examined using complementary empirical designs, including local incremental validity models with cross-validation, fairness analyses of comparability and predictive bias, decision-analytic simulations with base-rate and error-cost sensitivity, and policy evaluation designs for test-optional adoption, such as event studies and difference-in-differences (Osaki, 2022; Rosinger et al., 2024). Table 5 maps each proposition to concrete empirical strategies, key metrics, sample structures, and major threats to inference.

## 8. Conclusions

Intelligence testing predicts academic achievement, but predictive evidence alone does not determine whether cognitive measures should be used in educational decisions. The central issue is decisional: whether cognitive information changes outcomes in a meaningful way under explicit constraints of fairness, legitimacy, and institutional purpose. From this perspective, the relevant question is not whether intelligence tests are valid in the abstract but when their use is justified across selection, placement, diagnosis/eligibility, and intervention-planning decisions. The result is neither a blanket endorsement nor a categorical rejection of intelligence testing, but rather a principled case for its conditional and sequential use: begin with achievement, grades, and contextual evidence, add cognitive assessment only when it is likely to change the decision, and evaluate consequences in terms of both outcomes and distributional impact. In this way, the debate shifts from polarized claims about testing toward a more testable and context-sensitive account of when cognitive assessment improves educational decision-making.

### 8.1. Limitations

Because this is a theoretical article, we do not provide a single empirical estimate of decision utility. Instead, we specify mechanisms and boundary conditions under which incremental validity should translate, or fail to translate, into better decisions.

A further limitation concerns institutional scope and transferability. The framework is developed primarily with reference to North American and other Anglo-American assessment debates, especially postsecondary admissions, high-potential or gifted program placement, and SLD/ID-related evaluation. Although its core decision logic may travel across systems, its implementation will vary across institutional contexts. In systems with centralized admissions, national examinations, early tracking, different disability classification rules, or different legal definitions of fairness, the staged architecture would need to be recalibrated around local decision units, available evidence, policy constraints, and accountability regimes. Future work should examine how the architecture performs across different national and institutional systems.

In addition, some post-2020 evidence on test-optional regimes necessarily draws on working papers and institutional reports alongside peer-reviewed studies, reflecting rapid policy change and publication lag; these conclusions should be updated as more causal and multi-institution datasets accumulate. Finally, fairness and legitimacy are multidimensional and cannot be reduced to a single metric. Rather than attempting to eliminate value conflicts, the framework is designed to make trade-offs explicit so they can be debated, justified, and monitored transparently.

### 8.2. Practical Guidance

In practical terms, the framework suggests that intelligence testing should not be treated as a default educational input but as decision-specific evidence whose value depends on purpose, context, and constraints. Where the aim is to certify current curriculum mastery or applied performance, achievement measures and authentic assessment may be more appropriate than cognitive testing. Where the decision concerns selection, placement, or complex evaluative judgment under uncertainty, cognitive evidence is most defensible when it is added selectively and when it can plausibly change the decision beyond grades, achievement, and contextual information already available. The practical implication is therefore not that intelligence testing should always be used or always rejected but that its use should remain bounded by decision relevance, psychometric defensibility, fairness constraints, and ongoing institutional accountability.

## Figures and Tables

**Table 1:** Decision purposes in education and implications for evidence use.

Decision Purpose	Primary Objective	Typical Unit of Decision	Evidence Bundle (Recommended)	Dominant Error-Cost Concern
Ranking/Selection (admissions, scholarships)	Optimize prediction under capacity constraints	Applicant vs. applicant (scarce seats)	GPA/transcript + achievement; cognitive/aptitude test when incremental; context indicators (school opportunity); noncognitive if decision-relevant	False positives (admit underprepared) vs. false negatives (reject capable); plus distributional impact and legitimacy
Placement (high-potential, gifted, or advanced programs, tracks, remediation)	Person–environment fit (who benefits from program)	Student vs. program track (program requirements)	Achievement + curriculum exposure; targeted cognitive assessment for discordant cases; teacher/mentor evidence; context	False negatives can lock out opportunity; false positives can create mismatch and dilute resources
Diagnosis/Eligibility (SLD, ID, etc.)	Clarify learning profile and rule-in/rule-out	Student vs. criterion threshold (service eligibility)	Norm-referenced achievement; response to instruction/intervention; targeted cognitive testing when it changes conclusions	Misclassification (stigma, missed services); emphasis on interpretability/actionability
Intervention planning	Select supports that change outcomes	Student × support plan (dynamic)	Progress monitoring; curriculum-based measures; functional skill assessment; cognitive data only if actionable	Wrong intervention (time lost); failure to adapt supports to student context

**Table 2:** Stylized simulation of decision utility under two evidence regimes.

Scenario	Regime	Base Rate in Pool	Seats (K)	True Positives (TP)	False Positives (FP)	Precision (TP/K)	Net Benefit
A	Achievement-only	0.40	200	125	75	0.625	0.025
A	Achievement + test	0.40	200	139	61	0.695	0.058
B	Achievement-only	0.25	200	96	104	0.480	−0.042
B	Achievement + test	0.25	200	112	88	0.560	−0.005

* Net benefit is computed with N = 1000 and p_t_ ≈ 0.571. Values are illustrative and are intended only to show how the framework behaves under changing decision conditions.

**Table 3:** When intelligence testing is expected to add decision utility, plus minimum standards.

Context/Condition	Expected Incremental Value	Recommended Decision Design	Minimum Standards (Gatekeeping Checks)
Transitions (new demands) e.g., high school to college, track acceleration	High (past achievement may not generalize)	Staged: screen with GPA/achievement; add cognitive/aptitude test only when it can change selection/placement	Measurement comparability for key groups; predictive bias; monitor distributional impact over time
Uneven opportunity structures (grades reflect access)	Moderate-to-high (achievement may underrepresent capacity)	Use context + prior achievement first; targeted cognitive assessment for discordant or borderline cases	Explicit trade-offs: validity vs. equity; selection-error sensitivity analyses
Discrepant profiles (underachievement, twice-exceptionality)	High for explanation/placement (not necessarily for mastery)	Targeted cognitive assessment paired with norm-referenced achievement and response-to-instruction data	Do not use IQ as a sole gate; require convergent evidence and decision relevance
High cognitive-load programs (fast pace, cumulative)	Moderate (especially when seats are scarce)	Combine achievement + cognitive/aptitude; evaluate error costs explicitly	Transparency and appeal process; periodic re-validation in local context
Stable environments with strong achievement signals	Low (achievement already captures most variance)	Prefer mastery/achievement evidence; avoid blanket testing	If used, justify incremental value and show no unacceptable disparate impact
Mastery certification only (course pass/fail, grading)	Low (goal is current curriculum)	Use curriculum-aligned achievement and authentic assessment; cognitive testing typically not needed	Ensure reliability/standardization of scoring if comparing across settings
Test-optional regimes (admissions)	Context-dependent; may reduce information for some applicants (strategic withholding)	Model strategic disclosure explicitly; compare regimes with decision-utility lens; staged prompts/guidance where scores add contextual signal	Evaluate policy as a system: accuracy, errors, enrollment composition, retention; consider heterogeneous implementation

**Table 4:** Implementation checklist (if-then) for schools and universities.

If…	Then… (Operational Action)	Minimum Written Record/Evidence
you plan to use a cognitive test for a real decision	define the decision purpose (selection vs. placement vs. diagnosis/eligibility vs. intervention planning) and the objective function (prediction accuracy, access, support allocation, legitimacy).	1-page brief: purpose + objective + population + decision.
you already have GPA/grades + achievement + context	estimate local incremental validity of adding cognition (does it change prediction or decisions?).	Local model + validation (ideally cross-validation) + “adds value: yes/no” result.
incremental validity does not change decisions or costs outweigh benefits	do not add cognitive testing (avoid unnecessary cost/exposure).	Short technical note: explicit “do not use” criterion.
you decide to implement it	use a staged architecture (screening → targeted assessment → decision + monitoring).	Official process flowchart.
you are in Stage 1 (screening)	use broad indicators (transcript/GPA + achievement + context) for triage.	Triage rule + allowed variables list.
the case is uncertain, borderline, or discordant	move to Stage 2: use cognitive testing only if it could change the decision.	“Uncertainty” criterion (gray zones/cutoffs).
you are in Stage 3 (final decision)	formalize decision rules with explicit base rates, false-positive/false-negative costs, capacity constraints, and equity trade-offs.	Cost matrix + base rates + final rule.
the decision is selection with scarce seats (admissions, scholarships, limited programs)	prioritize incremental validity and explicitly state error-cost + equity trade-offs.	Documented “accepted trade-offs” + thresholds.
the decision is placement (high-potential, gifted, or advanced programs, acceleration, or advanced track)	prioritize person–environment fit and asymmetric costs (e.g., missed opportunity).	Placement criteria + safeguards for missed opportunity.
the decision is diagnosis/eligibility (SLD/ID, support eligibility)	prioritize interpretability and actionability; use cognition only if it changes hypotheses or classification conclusions.	Note specifying what diagnostic or eligibility decision changes.
the decision is intervention planning	prioritize actionable information for selecting supports; use cognitive data only if it changes the support plan.	Note specifying what support decision changes.
stakes are high or you compare across groups	require measurement comparability, including invariance evidence where relevant, before interpreting group differences.	Instrument technical documentation + comparability report.
you use scores to predict outcomes (performance, retention)	test predictive bias (over/under-prediction by group).	Fairness report + mitigation plan.
there is public concern (stigma, historical misuse)	implement transparency: purpose, rules, process, plus an appeals pathway.	Frequently asked questions + appeals protocol + response timelines.
the system is live	monitor predictive performance, distributional impact, and legitimacy; revalidate periodically and recalibrate rules.	Semiannual/annual dashboard + review triggers.
you operate under test-optional, test-blind, or test-required (universities)	treat it as a system: model strategic disclosure and compare regimes by utility (errors, composition, retention).	Policy evaluation with scenarios + sensitivity analyses.

**Table 5:** Operationalization of P1–P4: designs, metrics, samples, and threats.

Proposition	Primary Design(s)	Key Metrics	Unit/Sample	Key Threats and Mitigations
P1	Local validation across transition points (e.g., end of secondary → first-year postsecondary). Compare models within stable vs. novel environments; cross-validated incremental validity.	ΔAUC, ΔBrier, calibration slope/intercept; cost-adjusted net benefit across p_t_; sensitivity to range restriction.	Student-level cohorts across multiple years/institutions; outcomes: first-year GPA/persistence vs. within-school grades.	Range restriction and selective samples → report unrestricted/cohort-wide models; measurement non-equivalence across contexts → comparability checks (including invariance where appropriate).
P2	Heterogeneity designs: interact cognitive measures with opportunity indicators (SES, school resources, grading regimes); within-school or within-region comparisons; partial pooling across settings.	Incremental validity stratified by opportunity; calibration by subgroup; net benefit under policies targeting underachievement/twice-exceptionality.	Student-level, multi-school datasets with opportunity proxies; oversample under-resourced settings when possible.	Unobserved confounding in opportunity proxies → rich covariates/fixed effects; differential measurement quality → comparability and reliability checks.
P3	Experimental or quasi-experimental evaluation of staged vs. blanket testing: cluster RCT/stepped-wedge across schools, or simulation using real score distributions and decision rules.	Net benefit per unit cost; testing exposure; false positive/negative rates at operational margin; downstream outcomes (placement fit, support uptake).	School/district-level implementation units; students as observations. Compare Stage 2 targeted testing vs. universal testing.	Contamination and noncompliance → intention-to-treat + compliance analysis; changing base rates → recalibration; capacity constraints → explicitly model K and rule-based thresholds.
P4	Policy evaluation of test-optional/test-blind adoption: event study + DID with institution fixed effects; synthetic control for early adopters; analyze strategic disclosure and information loss.	Changes in score submission rates; predictive performance of admissions models; shifts in error distribution; subgroup access and graduation outcomes; net benefit under stated objectives.	Institution-by-cohort panel across multiple admissions cycles; applicants and admits as units depending on outcome.	Selection into adoption and concurrent reforms → pre-trends, robustness, policy controls; compositional shifts → reweighting; strategic behavior → model disclosure explicitly.

## Data Availability

The original contributions presented in this study are included in the article. Further inquiries can be directed to the corresponding author.

## Abbreviations

The following abbreviations are used in this manuscript:

GPA	Grade Point Average
SLD	Specific Learning Disability
ID	Intellectual Disability
DCA	Decision-Curve Analysis
AUC	Area Under the Receiver Operating Characteristic Curve
